# Nanorobotic Investigation Identifies Novel Visual, Structural and Functional Correlates of Autoimmune Pathology in a Blistering Skin Disease Model

**DOI:** 10.1371/journal.pone.0106895

**Published:** 2014-09-08

**Authors:** Kristina Seiffert-Sinha, Ruiguo Yang, Carmen K. Fung, King W. Lai, Kevin C. Patterson, Aimee S. Payne, Ning Xi, Animesh A. Sinha

**Affiliations:** 1 Department of Dermatology, University at Buffalo, Clinical and Translational Research Center, Buffalo, New York, United States of America; 2 Department of Electrical and Computer Engineering, Michigan State University, The Robotics and Automation Laboratory, East Lansing, Michigan, United States of America; 3 Department of Mechanical and Biomedical Engineering, City University of Hong Kong, Kowloon, Hong Kong; 4 College of Human Medicine, Michigan State University, East Lansing, Michigan, United States of America; 5 Department of Dermatology, University of Pennsylvania, 217A Clinical Research Building, Philadelphia, Pennsylvania, United States of America; University Hospital Hamburg-Eppendorf, Germany

## Abstract

There remain major gaps in our knowledge regarding the detailed mechanisms by which autoantibodies mediate damage at the tissue level. We have undertaken novel strategies at the interface of engineering and clinical medicine to integrate nanoscale visual and structural data using nanorobotic atomic force microscopy with cell functional analyses to reveal previously unattainable details of autoimmune processes in real-time. Pemphigus vulgaris is a life-threatening autoimmune blistering skin condition in which there is disruption of desmosomal cell-cell adhesion structures that are associated with the presence of antibodies directed against specific epithelial proteins including Desmoglein (Dsg) 3. We demonstrate that pathogenic (blister-forming) anti-Dsg3 antibodies, distinct from non-pathogenic (non-blister forming) anti-Dsg3 antibodies, alter the structural and functional properties of keratinocytes in two sequential steps - an initial loss of cell adhesion and a later induction of apoptosis-related signaling pathways, but not full apoptotic cell death. We propose a “2-Hit” model for autoimmune disruption associated with skin-specific pathogenic autoantibodies. These data provide unprecedented details of autoimmune processes at the tissue level and offer a novel conceptual framework for understanding the action of self-reactive antibodies.

## Introduction

Desmosomal junctions are specialized structures critical to cellular adhesion within epithelial tissues. Disassembly of these junctions (acantholysis) can occur following autoimmune attack. Pemphigus vulgaris (PV) is a prototypical organ-specific, potentially life-threatening human autoimmune disease characterized clinically by flaccid blister formation affecting the skin and mucous membranes. PV exhibits an intraepidermal split due to acantholysis of suprabasilar keratinocytes that occurs in the presence of autoantibodies to specific desmosomal proteins, primarily desmoglein (Dsg) 3, and in some cases anti-Dsg1 [1]. Anti-Dsg3 autoantibodies have been shown to induce acantholysis in cultured keratinocytes [2] and blister formation *in* *vivo* in neonatal mice [3]. Anti-Dsg 1 antibodies are found in approximately 40% of PV patients, and have also been linked to the development of Pemphigus foliaceus, a closely related but distinct autoimmune blistering skin disease, where they are sufficient to induce blister formation [4].

Although lesion development in patients with PV is generally associated with high titers of anti-Dsg3 autoantibodies, the precise molecular mechanisms by which autoantibodies direct the loss of cell-cell adhesion is not known. In particular, it is unclear if acantholysis is the direct result of structural changes at the keratinocyte cell surface that occur subsequent to autoantibody binding and/or is dependent upon functional changes within the cell. Three major hypotheses have been proposed regarding the mechanisms by which anti-Dsg antibody binding to the cell surface leads to acantholysis in PV: (i) “steric hindrance”, the direct inhibition of Dsg transinteractions [5], [6], (ii) depletion of desmosomal proteins from the keratinocyte surface [7], and (iii) initiation of signal transduction events that lead to altered desmosome assembly, cytoskeleton derangement, cell cycle alterations, and apoptosis [8]. However, to date, there is no conclusive model of antibody-mediated acantholysis, and the role of apoptosis is unsettled. While apoptotic phenomena have been observed in PV, there is considerable disagreement regarding its role in acantholysis. Some groups have shown that acantholysis can occur in the absence of apoptosis [9], and find that hallmarks of apoptosis, such as changes in nuclear morphology and cell death, are detectable only late and subsequent to acantholysis [8]. Others favor the theory that apoptotic signaling precedes acantholysis, but necessarily leads to apoptosis, and have termed this paradigm “apoptolysis” [10]. Moreover, there is now clear evidence that PV patients harbor both anti-Dsg3 antibodies that lead to blister formation (pathogenic) and anti-Dsg3 antibodies that do not lead to blister formation (*non*pathogenic) [2], [11]. While both pathogenic and nonpathogenic autoantibodies are capable of binding to Dsg3 molecules expressed within desmosomal bodies at the keratinocyte surface, there remains a mystery as to why they lead to different clinico-pathological outcomes.

To address these outstanding questions, novel experimental technologies and strategies are required. To date, it has been extremely challenging to study the morphology of cell junctions by standard light microscopy in living cells because of their small size (<1 µm) and complex structure [12]. While immunofluorescence [13] [14] and electron microscopy [15] have provided insight into the fine structure of cell adhesion molecules, a model system for addressing dynamic changes due to (patho-) physiological mechanisms has been lacking. Atomic force microscopy (AFM) offers the advantage of requiring minimal sample preparation, so that biomolecular structures can be directly studied *in* *situ* on viable samples that recapitulate biological conditions. AFM provides three-dimensional images of surface topography in unparalleled resolution allowing for the illumination of structural modifications of adhesion structures after antibody treatment at a scale that cannot be revealed by standard light microscopy and also provides quantitative measures of biological properties (e.g. cellular elasticity) in a physiologically stable environment.

In this study, we utilized established and novel roboticized AFM methods to visualize desmosomes in physiologic and disease conditions at the nanoscale, and to determine detailed nanostructural correlates of the acantholythic process not previously attainable. Furthermore, we undertook an innovative, interdisciplinary approach to link AFM data to functional alterations in cell behavior to develop a new paradigm for autoantibody mediated tissue destruction in the skin. We reveal new details regarding the molecular basis for the functional dichotomy between pathogenic vs. non-pathogenic autoantibodies. Blister-forming anti-Dsg 3 antibodies produce changes in cellular stiffness that are distinct from the changes induced by non-pathogenic antibodies. Both pathogenic and non-pathogenic autoantibodies induce an early, but incomplete, disruption of intercellular adhesion (“Hit 1”), but pathogenic antibodies alone lead to a later induction of apoptosis-related signaling (“Hit 2”). These data advance our understanding of autoimmune destruction and support future nanoscale clinical applications relevant to the diagnosis and treatment of disease.

## Materials and Methods

### Keratinocyte cultures and antibodies

For the studies presented here, we used the HaCaT cell line, a spontaneously transformed human adult skin keratinocyte line that maintains a near normal phenotype [16]. HaCaT cells recapitulate normal human differentiation behavior in vitro, particularly in terms of desmosomal kinetics [17]. Prior to experimental use, HaCaT cells were grown to confluence in DMEM medium (Gibco-Invitrogen, Carlsbad, Ca) supplemented with 10% fetal calf serum (Gemini Bio-products, West Sacramento, Ca) and 1% penicillin:streptomycin (10,000 U/ml:10,000 µg/ml; Gibco) at 37°C in a humidified atmosphere containing 5% CO_2_, and plated onto poly-L-ornithine (Sigma, St. Louis, MO) coated glass coverslips placed inside 12-well tissue culture plates.

We used the pathogenic anti-Dsg3 antibody Px4-3 or the non-pathogenic antibody Px4-4, single-chain variable-region fragment (ScFv) monoclonal antibodies isolated from a patient with mucocutaneous PV by phage display. The antibodies were diluted in phosphate buffered saline (PBS) or cell culture medium at 1∶50. Purified goat anti-mouse Ig antibody (BD Pharmingen, San Jose, CA) was used as an irrelevant non-binding control antibody, and mouse anti-human HLA-A, B, C antibody (BD Pharmingen) was used as a cell-surface binding control antibody, both at a 1∶50 dilution. To measure the induction or blocking of apoptotic processes, in some experiments, Fas Ligand (50 ng/ml), Fas Ligand neutralizing antibody (0.5 ng/ml), and/or caspase inhibitor (20 µM; all BD Pharmingen) was used.

### Immunofluorescence Microscopy

HaCaT cells were grown to confluence and fixed in 99.93% dry methanol for 5 minutes. Monoclonal murine anti-human cytokeratin peptide 18 antibody (Sigma, St. Luis, MO) was added at a 1∶100 dilution and goat anti-human desmoplakin antibody I/II (Santa Cruz Biotechnology, Inc, Santa Cruz, CA) was added at a 1∶400 dilution and the cells were incubated at 37°C for 30 minutes. After extensive washing with PBS, PE-conjugated goat anti-mouse IgG1 (Santa Cruz Biotechnology), or AF488-conjugated donkey anti-goat IgG antibodies were added at 1∶400 and 1∶200 dilutions for 30 minutes at 37°C to visualize cytokeratin or desmoplakin stains. After washing with water, polyvinyl alcohol mounting medium (Dabco, Sigma, St. Luis, MO) was applied. The immunofluorescence imaging was performed under Nikon Intensilight C-HGFI light source with G-2A filter.

### SEM imaging

HaCaT cells were fixed in a mixture of 2.5% glutaraldehyde and 2.5% formaldehyde in 0.1 M sodium cacodylate buffer for 2 hours at room temperature. After rinsing with the buffer only solution for 15 minutes, the sample went through a sequential dehydration process with 25%−, 50%−, 75%−, 95%− and 100% ethanol. The dehydrated sample was then dried inside a critical point drier (PELCO CPD2, Pelco International Redding, CA). The image was taken under 20 kv acceleration voltage in a Joel SEM (JEOL, Peabody, MA) after coating with a thin layer of gold.

### AFM imaging

For imaging living HaCaT cells at *low resolution*, a Bioscope AFM (Bruker AXS, Santa Barbara, CA), equipped with a scanner with a maximum XY scan range of 90 µm × 90 µm and a Z range of 5 µm, was used. For imaging living HaCaT cell at *high resolution*, a multimode AFM (Bruker AXS) was used. Experiments were performed in culture medium at room temperature using silicon nitride cantilevers with a spring constant of 0.38 N/m and mounted in a commercially available fluid cell module. All scans were completed by taking 256×256 point scans and recording topographic data. Both the trace and retrace images were measured and compared. For *fixed cell imaging* under antibody treatment, different types of antibody (pathogenic anti-Dsg3 antibody, non-pathogenic anti-Dsg3 antibody, non-binding irrelevant antibody and binding irrelevant antibody) were added to individual wells in DMEM medium for 24 h at a final antibody concentration of 10 µg/ml. Cell fixation was achieved by incubation in 3.7% formaldehyde (Sigma-Aldrich) for at least 15 minutes and visualized using the multimode AFM. For *live cell imaging* under antibody treatment, the antibodies were added without fixation at a final concentration of 10 µg/ml under continuous Bioscope AFM observation.

### Cell elasticity measurements

Force-displacement curves measurements were performed under a Nanoscope IV Bioscope (Bruker AXS). Silicon nitride tips with a spring constant of 0.06 N/m and a tip radius around 10 nm were used to probe the cells. An indentation speed of 1.8 µm (corresponding to a frequency of 0.3 Hz) was selected to reduce the cell viscous effect [18] but maintain a sufficient time frame to capture dynamic changes of cell properties. To ensure data reliability, all measurements were performed in the cell center. During this process, the indentation force was controlled at 10 nN and the indentation depth 500 nm. Collected force-displacement curves were processed with Matlab routines to convert them to force-indentation curves and then fitted with the Hertz model to generate the Young's modulus (E). The tip half opening angle is 17.5 degrees and the Poisson ratio is set as 0.5.

### Nanorobotic surgery

AFM-based nanorobotics enables accurate and convenient sample manipulation and drug delivery. This capability was used in the current study to control the AFM tip position over the intercellular junction area, and apply vertical indentation forces, so that bundles of intercellular adhesion structures can be dissected precisely with an accuracy of less than 100 nm in height. We used a tip sharp enough (2 nm in tip apex diameter) to penetrate the cell membrane and the intermediate filaments. It has been shown that intermediate filaments have extremely high tensile strength by *in* *vitro* AFM stretching [19]. Thus, the vertical force and moving speed of the AFM cantilever (0.06 N/m in vertical spring constant) was controlled at a vertical force of 5 nN at an indentation speed of 0.1 µm/s to guarantee the rupture of the filament and to partially dissect cell adhesion structures between two neighboring cells.

### Measurement of apoptosis and cell death by flow cytometry

HaCaT cell were grown to confluency as describe above. Two days after reaching confluency (to insure formation of strong intercellular adhesion structures), cells were treated with the respective anti-Dsg3 or irrelevant control antibodies for the indicated time periods before harvesting the cells by trypsinization. In some experiments, Fas Ligand (BD Pharmingen) was added 48 h before harvesting the cells. In experiments including Fas Ligand neutralizing antibody (BD Pharmingen) or caspase inhibitor (BD Pharmingen), these agents were added 30 min prior to adding Fas Ligand or the anti-Dsg3 antibody. Trypsinized cells were washed in PBS and combined with dead cells from their conditioned supernatants. To detect apoptotic and/or dead cells, HaCaT cells were re-suspended in staining buffer, and annexin V antibody and/or propidium iodide were added as per manufacturer's instructions (Annexin V-FITC apoptosis detection kit, BD Pharmingen). Apoptosis and cell death was analyzed by FACS on a Vantage SE (BD Biosciences) flow cytometer.

## Results

### Nanoscale visualization of keratinocyte junctions under physiologic and disease conditions

**Figure 1:**
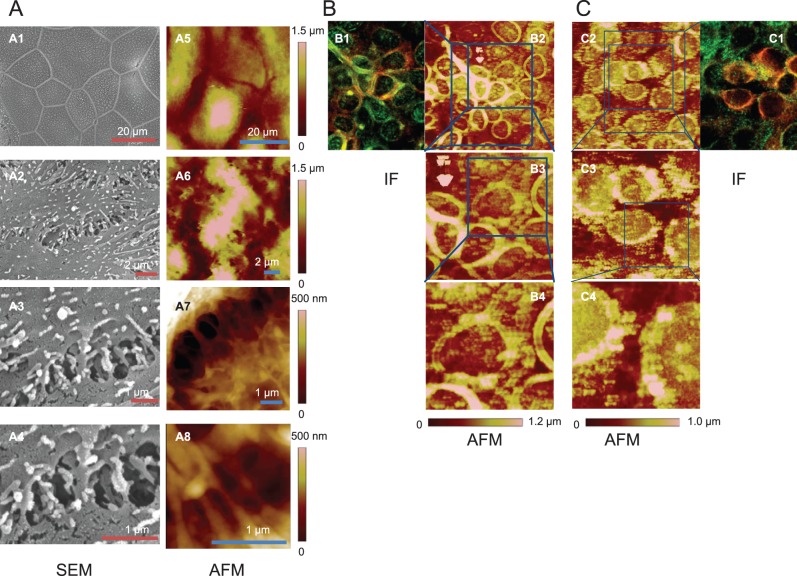
Integrated scanning electron microscopy (SEM), immunofluorescence (IF) and AFM imaging of intercellular adhesion structures. **A:** Correlation of SEM imaging with AFM imaging. Identically cultured plates of confluent HaCaT cells were fixed and imaged by SEM and Multimode AFM in increasing magnifications. The lower magnification images (SEM: **A1, A2;** AFM: **A5, A6**) show the cells with clear boundaries between neighboring cells where cell-cell adhesion occurs. The higher magnification images (SEM: **A3,** **A4;** AFM: **A7, A8**) show details of the adhesion junction with strand-shaped structures in parallel distribution between two cells. **B:** Correlation of IF imaging with AFM imaging. The same area on a confluent slide of HaCaT cells was captured simultaneously by IF and AFM after fixation of the cells. For IF imaging, HaCaT cells were labeled with anti-cytokeratin antibodies (red) and anti-desmoplakin antibodies (green). AFM images were captured by Bioscope AFM at increasing resolution with scan sizes of 100 µm (**B2**), 50 µm (**B3**) and 20 µm (**B4**). **C:** Correlation of IF imaging (**C1**) with AFM imaging [scan sizes of 100 µm (**C2**), 50 µm (**C3**) and 20 µm (**C4**)] after treatment with 10 µg/ml of the pathogenic anti-Dsg3 antibody Px4-3 for 24 h.

To validate the applicability of AFM for the investigation of cell-cell adhesion, we conducted high magnification AFM imaging of fixed HaCaT cells, a well-established keratinocyte cell line. We show that the AFM images correspond well with SEM imaging (considered the gold standard in high resolution imaging of detailed intercellular adhesion structures in previous work [20]) (**Fig. 1A**).

**Figure 2:**
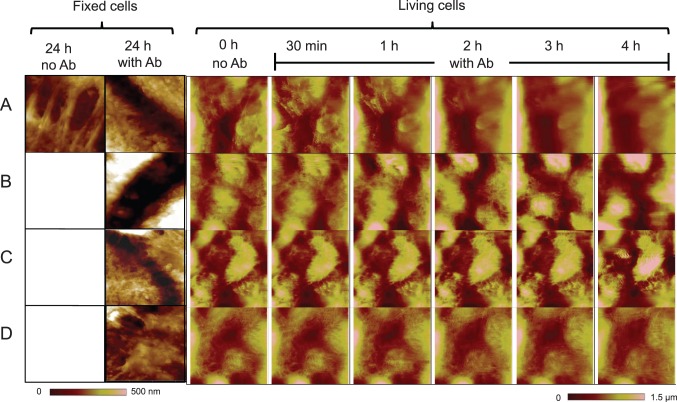
AFM imaging of autoantibody mediated desmosomal disruption in fixed and living cells. In a first set of experiments, HaCaT cells were grown to confluence, and left with no antibody treatment, or were treated for 24 h with (**A**) the pathogenic anti-Dsg3 antibody Px4-3, (**B**) non-pathogenic antibody Px4-4, (**C**) non-binding irrelevant control antibody goat anti-mouse Ig, or (**D**) irrelevant control antibody mouse anti-human HLA A, B, C (all antibodies at 10 µg/ml). Images were captured in high resolution (range 6–8 µm) by Multimode AFM *after fixation* of the cells. In a second set of experiments, confluent HaCaT cells were *imaged live* before antibody treatment or treated with the antibodies listed above (**A–D**) for 30 min, 1 h, 2 h, 3 h, and 4 h.

Next, we demonstrate a close correlation of AFM images with IF images that visualize cytokeratin, an intermediate filament protein, and desmoplakin, an anchoring protein that links desmosomal proteins to intermediate filaments (**Fig. 1B**). Autoantibody-treated cells (**Fig. 1C**) display a distinctive enlarged spacing between neighboring cells when compared to untreated cells (**Fig. 1B**), indicating cell dissociation that is clearly observed both by IF and AFM in fixed cells. In the higher resolution AFM images of untreated *fixed* keratinocytes (**Fig. 2A**), we observe structures in parallel organization between the cell membranes. However, in cells incubated with pathogenic anti-Dsg3 antibody or non-pathogenic anti-Dsg3 antibody for 24 h, these structures linking adjacent cells are no longer clearly detectable (**Fig. 2A and B**, respectively), indicating that Dsg3-specific antibody binding leads to a perturbation of normal cell-cell adhesion. Conversely, the addition of two disease-irrelevant antibodies (goat anti-mouse Ig and mouse anti-human HLA A, B, C) does not alter the intercellular structures observed without treatment (**Figure 2C and D**, respectively).

To visually follow disease processes in real-time, we obtained images of *living* keratinocytes in culture before and after treatment with the same test and control antibodies used above over a time-course of 30 min to 4 h. Prior to antibody treatment (0 h), multiple intact intercellular structures can be observed. After treatment with the blister-inducing pathogenic anti-Dsg3 antibody, an initial perturbation of cell-cell adhesion can be visualized between 30 m–1 h, with all cells completely disconnected after 2 h (**Fig. 2A**). Treatment with the non-pathogenic antibody, which does not lead to blister formation *in* *vivo*, results in a similar initial disruption of cell-cell adhesion structures as seen with the pathogenic antibody. However, the non-pathogenic antibody associated changes appear with a delayed onset and do not lead to a complete loss of cellular shape (**Fig. 2B**). Neither of the irrelevant control antibodies alters the intercellular structures between adjacent cells at the corresponding time points (**Fig. 2C and D**). These data demonstrate the facility of AFM technology not only to document autoantibody associated effects at static time points in fixed specimens, but also to track autoantibody mediated effects on intracellular adhesion in real-time.

### Nanorobotic methods define cell structural correlates of autoantibody binding

**Figure 3:**
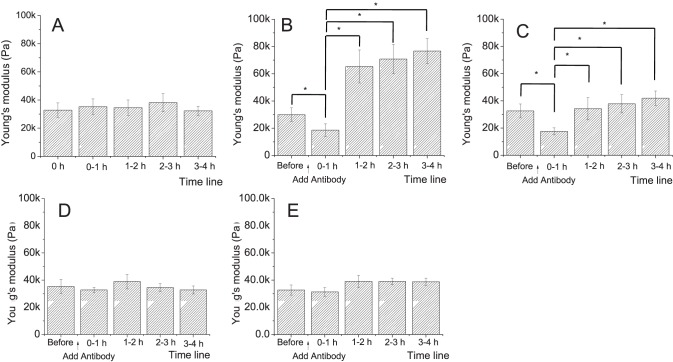
AFM-guided elasticity measurements on HaCaT cells before and after treatment with anti-Dsg3 and control antibodies. HaCaT cells were grown to confluence and elasticity was measured in the center of individual cells with an indentation speed of 1.8 µm/s (corresponding to a frequency of 0.3 Hz) over a 4 h time interval (**A**). At each time point, between 20 and 40 HaCaT cells were tested, and the force curves obtained from these measurements were processed to generate the corresponding Young's modulus as a measure of cellular elasticity. In parallel experiments, measurement were obtained before antibody treatment and after treatment with 10 µg/ml of the pathogenic anti-Dsg3 antibody Px4-3 (**B**), the non-pathogenic antibody Px4-4 (**C**), the non-binding irrelevant control antibody goat anti-mouse Ig (**D**), or the binding irrelevant control antibody mouse anti-human HLA A, B, C (**E**). Each bar represents the average and standard deviation of repeated measurements obtained over a 1 h period. Each graph represents the average of a minimum of 3 separate experiments for each antibody. The differences in cellular elasticity between conditions within 1 h after antibody treatment compared pre-antibody treatment, and 1–2 h, 2–3 h, and 3–4 h after antibody treatment, and were assessed by Student's t-test and are depicted as * = p≤0.05.

Dynamic mechanical properties of cells are increasingly being recognized as indicators and regulators of physiological processes [21]. For example, it has been shown that changes in cell elasticity/stiffness can effectively and reliably differentiate cellular conditions, such as non-cancerous vs. metastatic cells [18], [22], or the induction of apoptosis [21]. To obtain dynamic measurements under physiological and pathophysiological conditions, we continuously measured elasticity/stiffness changes over a 4-hour time course following antibody treatment of HaCaT cells. In untreated cells, or those treated with irrelevant control antibodies, the overall elasticity remained at the same level over the measurement interval of 4 hours with means ranging from 32 kPa to 36 kPa (**Fig. 3A, D, and E**, respectively). In the presence of the non-pathogenic antibody Px4-4, however, elasticity initially decreases within 1 h from baseline levels of 32.5±5.1 kPa to 17.6±2.6 kPa, then returns to baseline after 2 h, and remains between 35 kPa and 40 kPa thereafter, at similar levels to the initial value before antibody (**Fig. 3C**). In cells treated with the pathogenic antibody Px4-3, stiffness first drops at 1 hour to 18.6±4.6 kPa, similar to the results with non-pathogenic antibody. However, in contrast to the non-pathogenic antibody condition, at 2 h there is a consistent, sharp increase (∼three-fold) in stiffness to levels around 70 kPa for the remaining period (**Fig. 3B**).

To define the (patho-) physiological correlates of the early decrease in cell stiffness induced by both pathogenic and non-pathogenic antibody binding, we first investigated the extent to which the directed detachment of intercellular adhesion molecules via the manipulation of calcium levels in culture media (calcium depletion) or by nanorobotic surgical methods (nanodissection) affects elasticity measurements.

Desmosomal adhesion in vitro is calcium dependent. When the calcium concentration is raised, desmosomes form rapidly between cells cultured. However, the formation of cultured desmosomes can be interrupted by the depletion of calcium from the growth medium (below <0.1 mM) [23] due to the formation of half desmosomes that are subsequently internalized [24]. Calcium-depleted cells grow separately from their neighboring cells, or, if they had previously reached confluence, dissociate from adjacent cells.

**Figure 4:**
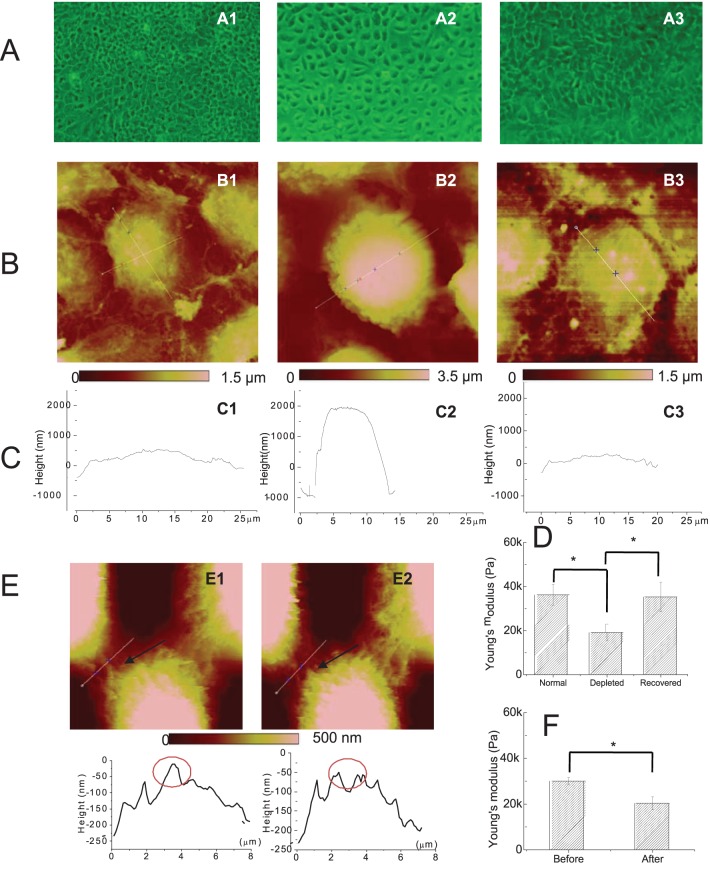
Decreased cell stiffness after dissociation of intercellular adhesion. HaCaT cells were cultured in calcium-containing medium for 4 days until reaching confluence (**A1, B1, C1**), and then calcium-depleted for 2 days (**A2, B2, C2**), or calcium-depleted for 2 days and then reconstituted in calcium-containing medium for another 2 days (**A3, B3, C3**). At each interval, we obtained (**A**) microscopic images (original magnification: 100x), (**B**) AFM topography images (scan size **B1**, 50 µm; **B2**, 20 µm; **B3**, 33.3 µm), and (**C**) height measurements at the cross section of an individual cell. In parallel, AFM-guided cell stiffness measurements were obtained by AFM nanomechanical measurement of 50 different cells under each of the conditions described above (**D**); Each bar represents the average and standard error of the mean (SEM) of repeated measurements for each condition. In a second set of experiments, cell adhesion structures in confluent HaCaT cell cultures grown under standard conditions were visualized by AFM prior to (**E1**) and after (**E2**) dissection of cell adhesion structures by nanorobot-guided surgery (traveling distance of AFM tip ∼8 µm; scan size: 19.2 µm; upper panel). The corresponding cross section height measurements show a height decrease by slightly less than 100 nm (from ∼10 nm to −100 nm in the encircled area; lower panel); the arrow in the AFM images points to an intact intercellular connection before cutting (**E1**) and the disappearance thereof after cutting (**E2**). **F**. Cell stiffness was measured before and after the nanorobotic surgery and decreases from 30.0±1.5 kPa to 20.5±2.8 kPa, respectively. The bars represent the mean and standard error of the mean (SEM). **E and F**. The differences in cellular elasticity between conditions before and after Ca+-depletion and recovery and before and after nano-dissection were assessed by Student's t-test and are depicted as * = p≤0.05.

We show that cells grown in calcium-containing medium form stable cell attachments to their neighboring cells, exhibit a somewhat “flat” appearance (diameter ∼25 µm, height ∼1 µm), and intercellular filaments bridging neighboring cells are clearly observed (**Fig. 4A** **1, -B1, -C1**). Calcium depletion leads to a loss of cell-cell adhesion that can be observed microscopically as an enlarged spacing between cells, and via AFM by a disappearance of intercellular filaments (**Fig. 4A** **2** and **B2**). Additionally, calcium depleted cells become approximately three times smaller in diameter (∼10 µm), but almost three times higher (∼3 µm) (**Fig. 4C** **2**). Once Ca^2+^ containing medium is reconstituted to cultures, cells recover to normal pre-depletion shape (**Fig. 4A** **3, -B3, -C3**). AFM-measurements of cellular stiffness at the 3 states (pre-depletion, post-depletion, and recovery) clearly show that the loss of intercellular adhesion, and thus change in cell shape, is tightly correlated with a decrease in cellular stiffness (from 36.5±4.7 kPa to 19.1±3.9 kPa). The re-formation of intercellular adhesion, however, restores stiffness back to levels seen before the loss of cell-cell connections (35.2±6.7 kPa) (**Fig. 4D**).

As an alternative method of disrupting intercellular adhesion, we performed novel, targeted nanodissection at the intracellular adhesion junction of keratinocytes using an AFM based nanorobotic surgery system. The AFM topography image (**Fig. 4E**) shows three keratinocytes connected by intercellular strands. Prior to nanodissection, the height of the cell junction was recorded. After nanodissection, a loss of intercellular strands is observed in the AFM images (see **Fig. 4E** **2**, black arrow), and is associated with a reduction in junction height by less than 100 nm. This process was also associated with a decrease in diameter and increase in cellular height of cells adjacent to the nanodissected area similar to the changes in cell shape observed by Ca+-depletion above (data not shown). Corresponding AFM measurements of cellular elasticity indicate that the stiffness of an individual HaCaT cell decreases from 30.0±1.5 kPa before to 20.5±2.8 kPa after nanodissection (**Fig. 4F**).

### Functional correlates of nanostructural alteration associated with autoantibody binding

To define the (patho-) physiological correlates of the increase in cell stiffness induced by pathogenic-, but not non-pathogenic antibody binding after the initial decrease in cell stiffness described above, we next investigated whether the internal remodeling of the cytoskeleton in cells undergoing apoptosis is reflected in dynamic changes in cell structural properties measurable by AFM, as has been suggested [21]. While the morphological characteristics typically associated with apoptosis are not necessarily detected in the histopathology of PV lesions, increased expression of apoptotic signaling molecules has been found in both PV lesions and patient serum when compared to healthy controls, and PVIg can trigger the activation of apoptotic signaling pathways in vitro [8]. Thus, we investigated whether the late increase in cell stiffness detectable at 2 h after pathogenic antibody treatment has its pathophysiological correlate in an induction of apoptotic processes by examining the extent to which (i) apoptosis is induced by pathogenic anti-Dsg3 antibodies, (ii) similar levels of cell stiffness increases can be observed with established inducers of apoptosis, and (iii) the antibody induced stiffness increase can be reversed by blocking apoptotic signaling.

**Figure 5:**
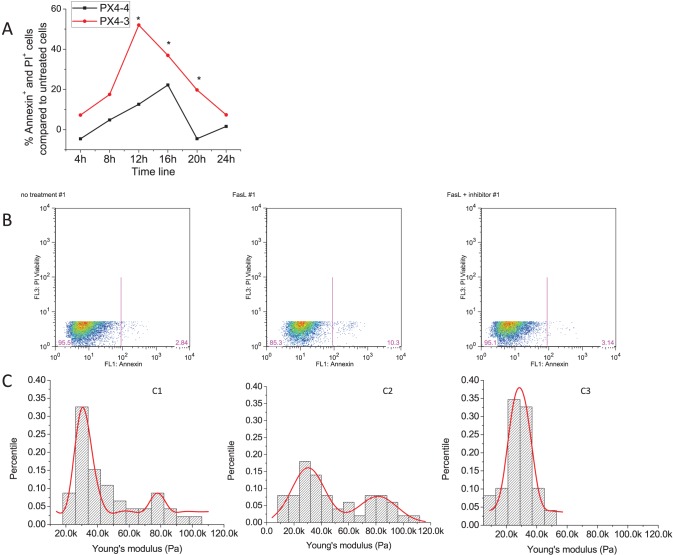
A. Induction of apoptotic processes by pathogenic anti-Dsg3 antibodies. Confluent HaCaT cell cultures were treated with 10 µg/ml of either the pathogenic anti-Dsg3 antibody Px4-3 or the non-pathogenic anti-Dsg3 antibody Px4-4 for 4, 8, 12, 16, 20 and 24 h. Induction of apoptosis was assessed by measuring the expression of both Annexin V and PI positivity by FACS. Each experiment was performed in triplicate. Values are presented as percent induction when compared to baseline apoptosis levels in untreated cells harvested in parallel. The levels of annexin V and/or PI positivity induced by pathogenic anti-Dsg3 antibodies are significantly elevated at 12, 16 and 20 h when compared to untreated cells (p≤0.05, Student’s t-test). **B and C. Fas ligand-induced apoptosis correlates with a marked increase in keratinocyte stiffness.** Subconfluent HaCaT cell cultures were left untreated (**B1**), treated with 50 ng/ml FasL (**B2**) or pretreated with FasL neutralizing antibody (0.5 µg/ml) for 30 min before addition of 50 ng/ml FasL (**B3**). In untreated HaCaT cells, 4.52±0.32% of all cells are Annexin V- and PI positive as measured by flow cytometry after 44 h of culture. Addition of FasL significantly increases the number of apoptotic cells to 14.63±0.11% (p<0.01, FasL vs. untreated). Pretreatment of FasL-treated cultures with FasL neutralizing antibody significantly reduces FasL-induced apoptosis back to baseline levels 5.95±1.28% (p<0.01, FasL + FasL inhibitor vs. FasL alone). In parallel experiments, 100 cells were randomly selected to collect AFM force-displacement curves to generate a Young's modulus value for each cell. The combined Young’s modulus values for each condition were plotted as Gaussian distribution curves. (**C1**) Normal cell stiffness distribution in untreated cells (Gaussian fit with peaks around 31.2 kPa and 77.8 kPa). (**C2**) Cell stiffness distribution in Fas ligand-treated cells (Gaussian fit with peaks around 31.5 kPa and 81.7 kPa). (**C3**) Cell stiffness distribution in Fas ligand treated cells pretreated with Fas ligand neutralizer (Gaussian fit with single peak around 29.1 kPa). Data are representative of three independent experiments performed in duplicate.

We first measured whether pathogenic anti-Dsg3 antibodies induce an activation of apoptotic pathways in cultured keratinocytes. In cells treated with pathogenic anti-Dsg3 antibodies, we observe an increase in combined Annexin V and PI positivity starting at 4 h, with a peak at 12 h and a return to baseline levels at 24 h (**Fig. 5A**). The non-pathogenic antibody induces a slight increase in Annexin V and PI positivity as well, but this increase does not reach significant levels at any time point, peaks later (around 16 h) and returns to baseline levels earlier (at 20 h) (**Fig. 5A**).

To verify that the sustained increase in cellular stiffness observed after pathogenic anti-Dsg3 antibody binding can also be found as a result of apoptosis induction, we used the well-established apoptosis-inducer Fas ligand (FasL). Addition of FasL significantly increases the number of apoptotic HaCaT cells in culture, while pretreatment of FasL-treated cultures with FasL neutralizing antibody significantly reduces FasL-induced apoptosis back to baseline levels (**Fig. 5B**). Parallel AFM force measurements show that untreated HaCaT cells and Fas ligand treated cells have a low peak in their stiffness distribution around 30 kPa and a high peak around 80 kPa. In FasL treated cells the high stiffness peak sharply increases (28% of all cells, **Fig. 5C** **2**) when compared to untreated cells (12%, **Fig. 5C** **1**). When apoptosis is blocked by FasL neutralizing antibody, the second peak of higher stiffness completely disappears (**Fig. 5C** **3**). These gain of function and loss of function data indicate that the induction of apoptosis in HaCaT cell cultures leads to a sharp increase in cell stiffness. However, our observation of the complete disappearance of cells displaying high stiffness in the presence of FasL inhibitor while low levels of background apoptosis remain detectable by FACS suggests that some FasL-independent apoptosis may have been induced before addition of FasL inhibitor. This baseline apoptosis can still be detected by FACS after 44 h of culture, while AFM detects the complete blockage of FasL-dependent apoptotic signaling mechanisms in this experimental set-up.

**Figure 6:**
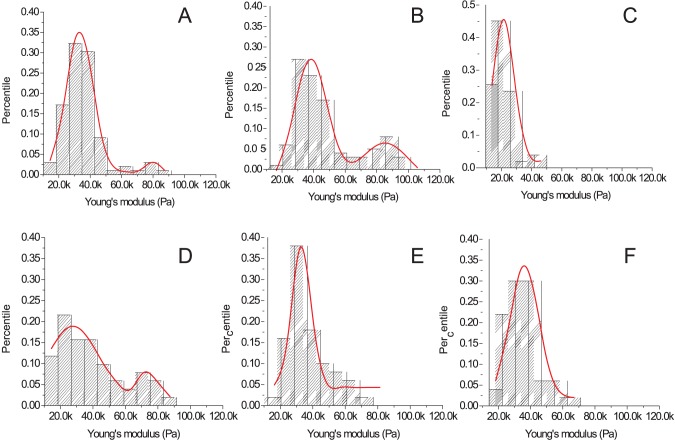
Blocking of pathogenic anti-Dsg3 antibody-induced increase in keratinocyte stiffness by caspase inhibition, but not Fas ligand neutralization. HaCaT keratinocytes were grown to confluence and cell stiffness was measured by AFM (expressed as percent of cells measured at a given Young’s Modulus). Cell stiffness distribution in (**A**) untreated cells (Gaussian fit with peaks around 32.0 kPa and 75.2 kPa), (**B**) cells treated with the pathogenic anti-Dsg3 antibody Px4-3 (10 µg/ml) alone for 8 h (peaks around 34.8 kPa and 83.5 kPa), (**C**) cells pretreated with caspase inhibitor (20 µM) 30 min before addition of pathogenic antibody (single peak around 17.7 kPa), (**D**) cells pretreated with Fas ligand neutralizing antibody (0.5 µg/ml) 30 min before addition of pathogenic antibody (peaks around 29.1 kPa and 73.0 kPa), (**E**) treated with caspase inhibitor (20 µM) alone for 8.5 h (single peak 30.1 kPa), or (**F**) treated with Fas ligand neutralizing antibody (0.5 µg/ml) alone for 8.5 h (single peak 32.3 kPa. The single peak of cells of low stiffness observed for treatment with caspase inhibitor (**E**) and FasL neutralizing antibody (**F**) alone indicates that the spontaneous apoptosis seen in untreated cells can be completely abrogated by these two blockers.

To investigate the extent to which the secondary increase in cellular stiffness produced by pathogenic autoantibodies can be linked to the induction of apoptosis, we blocked apoptosis in pathogenic anti-Dsg3 antibody treated cells by (a) inhibiting all caspases, a family of cysteine proteases that play essential roles in programmed cell death, or by (b) blocking the Fas/FasL pathway. Compared to baseline (**Fig. 6A**), addition of Px4-3 pathogenic antibody leads to a sharp increase in the number of cells with higher stiffness (22.5%, **Fig. 6B**) that is similar to the distribution of cell stiffness (both in terms of percentage of cells showing cellular stiffening as well as Young’s modulus values) as treatment with FasL (see **Fig. 5C** **2**). Pre-treatment of HaCaT cells with caspase inhibitor prior to the addition of Px4-3 antibody completely abrogates the development of cells with a stiffer profile, even below background levels (**Fig. 6C**), indicating that anti-Dsg3 antibody-induced apoptotic processes and blocking thereof can be monitored by AFM stiffness measurements. Pre-treatment of HaCaT cells with FasL neutralizing antibody before addition of Px4-3 antibody, however, does not reduce the number of stiffer cells (**Fig. 6D**). The disparate findings regarding the blocking of caspases or the FasL pathway indicate that while inhibition of apoptotic processes can reverse pathogenic autoantibody mediated phenomena, they are not mediated by the Fas/FasL signaling pathway. Independent of the stiffness induced by the pathogenic anti-Dsg3 antibodies, a small amount of cultured epithelial cells spontaneously display a stiffer profile that is amenable to blockage by both FasL- as well as caspase inhibition.

### 2-Hit hypothesis of anti-Dsg3 autoantibody mediated pathology in PV

**Figure 7:**
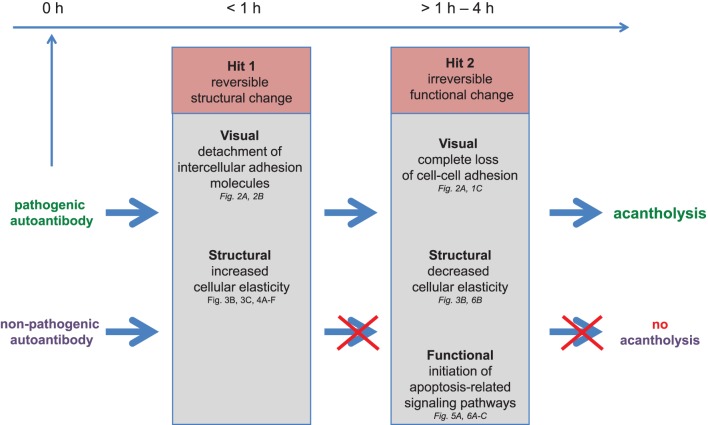
Two-Hit hypothesis for antibody-mediated damage in the skin. Pathogenic and non-pathogenic antibodies have distinct effects on keratinocyte biology: “Hit 1” reflects structural changes in the keratinocyte that are induced both by pathogenic and non-pathogenic antibodies within 1 hour with the pathophysiological correlate of an initial, but incomplete cellular dissociation. “Hit 2” involves a functional change in keratinocyte biology that is promoted by pathogenic, but *not* non-pathogenic antibodies within 1–4 h with the pathophysiological correlate of an initiation of apoptosis-related signaling pathways. Since the non-pathogenic antibody leads to an initial cellular dissociation that is also seen with pathogenic antibody binding, but does not alter the overall shape of the cell and does not induce the later cell structural changes seen with pathogenic antibodies, it follows logically that “Hit 2” is needed to progress to clinical disease, while “Hit 1” may be a reversible phenomenon.

Based on the AFM imaging and mechanical data as well as the functional experiments presented above, we propose a novel “**2-Hit hypothesis**” of anti-Dsg3 autoantibody effects in PV (**Fig. 7**). “Hit 1” reflects structural changes in the keratinocyte that are induced both by pathogenic (blister-forming) and non-pathogenic (non blister-forming) antibodies. “Hit 2” involves a functional change in the keratinocyte biology that is promoted by pathogenic, but *not* non-pathogenic antibodies. In this model, “Hit 1” has the pathophysiological correlate of an initial, but incomplete cellular dissociation, while “Hit 2” correlates with the initiation of apoptosis-related signaling pathways.

## Discussion

While AFM has been previously used to study the binding properties of anti-Dsg1 and 3 antibodies to planar surfaces in a cell-free environment [6], [25], we have applied novel nanorobotic AFM technologies for the investigation of autoimmune pathomechanisms *in* *vitro*. We show that, in addition to visually tracking the effects of PV-associated anti-Dsg3 autoantibody binding on cellular adhesion in high resolution and real-time, AFM can reliably detect nanostuctural changes at the intact cell surface in response to pathogenic (blister forming) and non-pathogenic (non blister forming) antibody binding and downstream signaling events that allow conclusions regarding disease pathomechanisms relevant to autoimmunity. Specifically, our experiments indicate that an early decrease in cellular stiffness detected by AFM after binding of both pathogenic as well as nonpathogenic anti-Dsg3 antibodies is consistent with a disruption of cellular adhesion, while a later increase in cellular stiffness induced by pathogenic, but not non-pathogenic antibodies may be the correlate of initiation of apoptosis-related signaling.

Both the calcium depletion as well as the nanorobotic manipulation experiments indicate that a disruption of intercellular adhesion leads to a dependable decrease in cellular stiffness, and are consistent with the initial decrease in cell stiffness measured by AFM within one hour after addition of autoantibody. Our data fits well with reports in the literature that see early effects of PV IgG on cell adhesion [26], [27] and cytoskeleton disassembly [28] within 30 min to 2 h. While significant Pemphigus IgG induced acantholysis usually takes 12–24 h to occur (reviewed in [26]), complete loss of cell adhesion has been observed within 1–6 h after antibody treatment with *pathogenic* antibody in experimental models [29].

Our *in* *vitro* apoptosis and caspase inhibition experiments indicate that an initiation of apoptosis-related signaling leads to a consistent increase in cell stiffness, and are consistent with the secondary, longer lasting increase in cell stiffness that is induced by pathogenic, but not non-pathogenic anti-Dsg3 antibodies beginning at 1–2 h after addition of antibody. We show that pathogenic antibodies induce a significant externalization of phosphatidylserine (PS) (as measured by Annexin V binding in this study), a common and accepted method for detecting apoptosis [30]. However, the return to baseline levels within 24 h after pathogenic antibody treatment indicates that the cells, while exhibiting apoptosis-related phenomena, have not undergone terminal apoptosis. Our data support previous findings by Pelacho et al. [31] that showed nuclear changes characteristic of apoptosis, with a clear peak after an 8 h incubation with PV-IgG, but a decrease thereafter. Contrary to long held beliefs regarding the irreversible fate of apoptotic cells, it has recently been shown that the initiation of apoptosis can be reversible with only transient exhibition of morphological signs of apoptosis at the cellular level [32]. Additionally, phosphatidylserine (PS) externalization (as measured by Annexin V binding in this study) is not restricted to apoptotic cells but has also been described for human CD8 cytotoxic T lymphocytes that reversibly expose PS upon T-cell receptor mediated antigen recognition [33]. Thus, similar to the reversible PS exposure on T cells upon antigen recognition, the reversible PS externalization on keratinocytes seen in our study upon autoantibody binding may indicate an initiation of apoptosis-related signaling cascades that has the potential to, but does not necessarily progress to apoptotic cell death in all conditions.

An activation of caspases has been observed in PV model systems, and caspase inhibitors have been shown to block PV IgG-induced acantholysis in keratinocyte monolayers and skin organ culture [34], [35]. Our finding that a caspase inhibitor can completely abrogate anti-Dsg3 induced changes in cell stiffness supports the notion that apoptosis-related signaling mechanisms are indeed involved in the downstream processes after antibody binding. However, as stated earlier, apoptosis-related signaling in PV may not necessarily progress to cell death, but exert its influence elsewhere, such as by targeting proteins involved in the regulation of cell contacts and of the cytoskeleton during apoptosis [36]. As such, caspase 3 has been shown to specifically cleave the cytoplasmic tail of Dsg3 and help release its extracellular domain from the cell surface, resulting in the disruption of the desmosome structure and contributing to cell rounding and disintegration of the intermediate filament system [35]. Along these lines, our ability to block anti-Dsg3 induced cell stiffening by blocking caspases could simply reflect an inhibition of Dsg3 cleavage, and not apoptosis. However, in keeping with our own data on the disruption of i.c. adhesion structures presented above, a disintegration of the keratin network would be expected to lead to cell softening rather than stiffening. Thus, it is conceivable that the anti-Dsg3 induced cell stiffening observed by AFM in this study can be viewed as a sensitive marker for the early initiation of apoptosis-related signaling pathways that may contribute to cellular dissociation, but not necessarily reach full apoptotic cell death. It is important to note that auto-antibody induced changes in cell topography and elasticity were detected by AFM at much earlier time points (starting at 30 min) than changes measured by standard flow cytometry assays (starting at 4 h), which suggests that AFM is a much more sensitive technique that allows for the detection of structural changes that precede downstream changes in cellular biology. Future studies into early signs of cell dissociation (such as retraction of keratin filaments) and the induction of early apoptotic and non-apoptotic signaling processes (caspase activation, p38 MAPK phosphorylation) will have to be correlated with the changes in cellular elasticity we observed in this paper in order to prove a direct causal relationship between these phenomena.

The inability of FasL neutralizing antibodies to reverse the pathogenic antibody-induced increase in stiffness is somewhat surprising, as an activation of the Fas-FasL apoptotic pathway has repeatedly been implicated in PV pathomechanisms [37], [38]. However, previous studies relied on observations from biopsy materials or *in* *vitro* studies with patient sera or purified IgG. It has been shown that sera from untreated PV patients contains high levels of FasL [37], which could conceivably lead to apoptosis independent of the effect of anti-Dsg antibodies. This notion is supported by the fact that inhibition of FasL or caspase 8 can only partially inhibit PV sera induced apoptosis [37]. In addition to containing FasL, serum autoantibody profiles vary among patients and may contain autoantibodies to targets other than Dsg [39], whose effects have not been well studied. In this study, we used a well-characterized and reproducible source of pathogenic PV anti-Dsg mAbs [11], [29] with non-pathogenic PV anti-Dsg mAbs as controls, in order to discern effects that can be attributed solely to the action of anti-Dsg3 on its cellular target.

The data presented in this study support the notion that a dissociation of cell adhesion structures precedes the detection of apoptosis-related signaling phenomena after pathogenic antibody attack. Based on the AFM imaging and mechanical data as well as the functional experiments presented here, we propose a novel “**2-Hit hypothesis**” of anti-Dsg3 autoantibody effects in PV (**Fig. 7**). Our hypothesis offers a new paradigm to explain why certain circulating autoantibodies lead to clinically overt blister formation on mucosal and cutaneous surfaces (i.e. pathogenic antibodies), while others do not result in blisters despite targeting the same autoantigen (i.e. non-pathogenic antibodies). It appears that simply interfering with desmosomal adhesion (either by steric hindrance or inducing depletion of desmosomal proteins from the cell surface as hypothesized before) is not sufficient to induce PV pathology, but that there is a requirement for this initial step to be followed by a secondary signaling event that ultimately induces acantholysis. This secondary signaling event appears to incorporate apoptosis-related phenomena, but does not require terminal cell death. This secondary signaling event could conceivably include p38 MAPK signaling, which has been suggested to be downstream of the loss of intercellular adhesion in PV [40]. Interestingly, a biphasic activation of p38 MAPK has been observed in response to Pemphigus foliaceous (PF) IgG, and the later peak has been suggested to lead to the activation of apoptotic pathways, but only lead to overt apoptosis in a small number of cells [41]. It remains to be determined whether the cell structural phenomena detected by AFM after pathogenic antibody binding are linked to MAPK activation and other intracellular events. Future studies are also needed to determine whether the dichotomy in autoantibody effects is related to the relative “strength” of the autoantibody (it is theoretically possible that the presumed “non”-pathogenic antibody does hold pathogenic potential at much higher concentrations per target cell not tested here), or to binding of different epitopes within the extracellular domains of the target molecule (such as in the amino-terminal region of Dsg [42]) which may be influenced by the 3D nature of intact tissue.

We demonstrate that AFM is an exquisitely sensitive method to detect cellular mechano-structural changes at a higher resolution and at earlier time-points than standard techniques. Advances in this technology allowed us to assign the sequence of specific nanostructural and functional changes consequent to the binding of pathogenic vs. non-pathogenic antibodies, and define critical pathways and processes responsible for autoimmune pathology. This information may facilitate predictive, nanoscale screening of individuals harboring disease relevant autoantibodies. The ability to distinguish the subset of autoantibody specificities within individuals that are disease causing (pathogenic) vs. those that are not could identify patients at risk for autoimmune progression and prioritize the key autoantibodies/antibody producing cells for therapeutic targeting in a given patient. We further envision the development of nanoscale devices designed to validate the in situ effectiveness of existing and emerging therapies. AFM-guided detection of mechanostructural changes in cells targeted by autoimmune processes has the potential to be applied to other models of autoimmunity in order to functionally investigate biological pathways and blockage of those pathways with potential novel and personalized therapeutic agents.
